# Detection of novel tick-borne pathogen, Alongshan virus, in *Ixodes ricinus* ticks, south-eastern Finland, 2019

**DOI:** 10.2807/1560-7917.ES.2019.24.27.1900394

**Published:** 2019-07-04

**Authors:** Suvi Kuivanen, Lev Levanov, Lauri Kareinen, Tarja Sironen, Anne J. Jääskeläinen, Ilya Plyusnin, Fathiah Zakham, Petra Emmerich, Jonas Schmidt-Chanasit, Jussi Hepojoki, Teemu Smura, Olli Vapalahti

**Affiliations:** 1 Department of Virology, University of Helsinki, Helsinki, Finland; 2 Department of Veterinary Biosciences, University of Helsinki, Helsinki, Finland; 3 Departments of Virology and Arbovirology, Bernhard Nocht Institute for Tropical Medicine, Hamburg, Germany; 4 University of Rostock, Rostock, Germany; 5 German Centre for Infection Research (DZIF), Hamburg, Germany; 6 Institute of Veterinary Pathology, Vetsuisse Faculty, University of Zurich, Zurich, Switzerland; 7 Division of Clinical Microbiology, University of Helsinki and Helsinki University Hospital, Helsinki, Finland; 8 Authors contributed equally

**Keywords:** Alongshan virus, ALSV, Jingmen tick virus, flavivirus, *Ixodes ricinus*

## Abstract

The newly identified tick-borne Alongshan virus (ALSV), a segmented Jingmen virus group flavivirus, was recently associated with human disease in China. We report the detection of ALSV RNA in *Ixodes ricinus* ticks in south-eastern Finland. Screening of sera from patients suspected for tick-borne encephalitis for Jingmen tick virus-like virus RNA and antibodies revealed no human cases. The presence of ALSV in common European ticks warrants further investigations on its role as a human pathogen.

Recent reports have associated two members of Jingmen virus group, Alongshan virus (ALSV) and Jingmen tick virus (JMTV), to febrile disease in humans [1,2]. Here we report the presence and genetic characterisation of ALSV in *Ixodes ricinus* ticks in Kotka archipelago, south-eastern Finland.

In 2010, a novel segmented tick-borne RNA virus, JMTV, was detected in *Rhipicephalus microplus* ticks in Hubei Province, China [3]. Subsequently, similar viruses have been identified in *R. microplus* and cattle in Brazil, i.e. the Mogiana tick virus (MGTV) [4-6]; human Crimean-Congo haemorrhagic fever (CCHF) cases in Kosovo* [7]; *Amblyomma javanense*, *Dermacentor silvarium* and *I. persulcatus* ticks as well as humans in China [1]; and a red colobus monkey in Uganda [8]. Recent reports associate novel JMTV strains from China with human disease [1,2]. A retrospective study conducted by Jia et al. reported identification of JMTV from skin biopsies and blood of febrile patients [1]. Meanwhile, ALSV was detected from *I. persulcatus* and isolated from febrile patient sera in Heilongjian Province [2]. These viruses share the genome organisation of four segments, two of which show similarity to the NS3 and NS5 proteins of non-segmented RNA viruses in the genus *Flavivirus*. The other two segments appear to originate from an unknown ancestor. Together, the viruses form a separate and diverse group tentatively called the Jingmen virus group in the family *Flaviviridae* [9].

## Detection of Jingmen-like virus in Kotka archipelago

**Figure 1:**
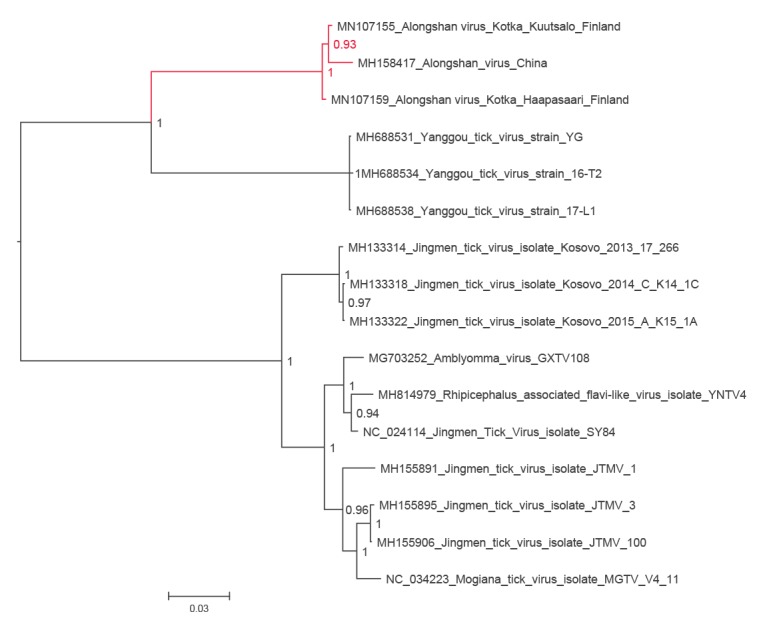
The phylogenetic tree of NS3 segment of JMTV-like viruses

**Figure 2:**
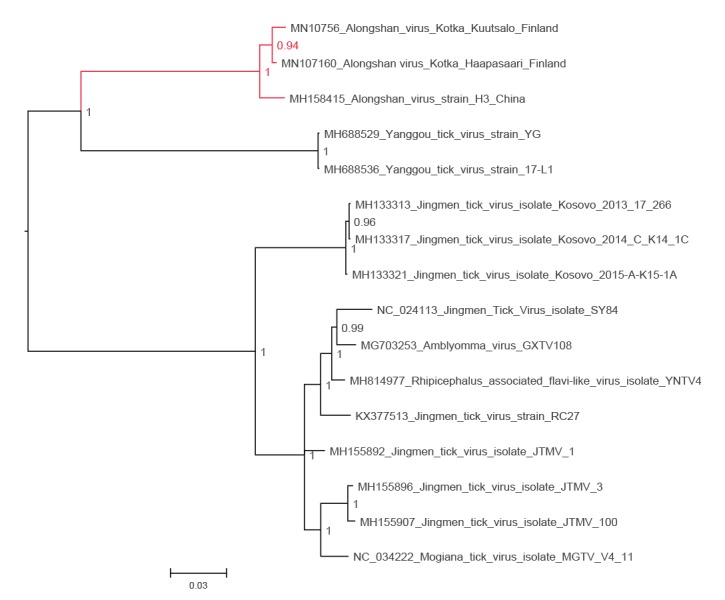
The phylogenetic tree of NS5 segment of JMTV-like viruses

**Figure 3:**
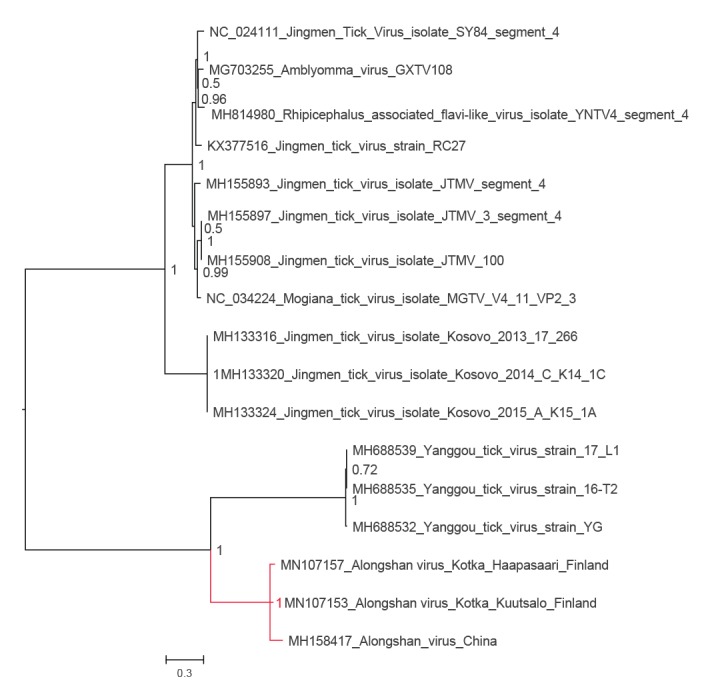
The phylogenetic tree of putative capsid/membrane segment of JMTV-like viruses

**Figure 4:**
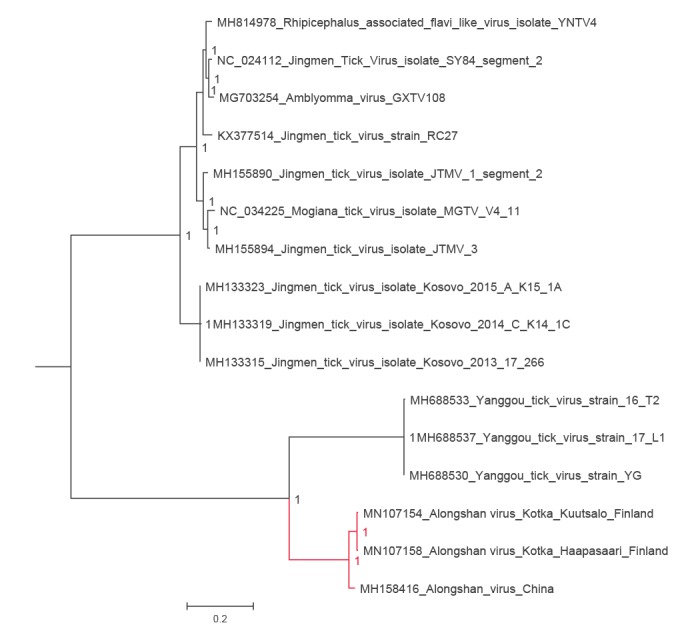
The phylogenetic tree of putative glycoprotein segment of JMTV-like viruses

**Figure 5:**
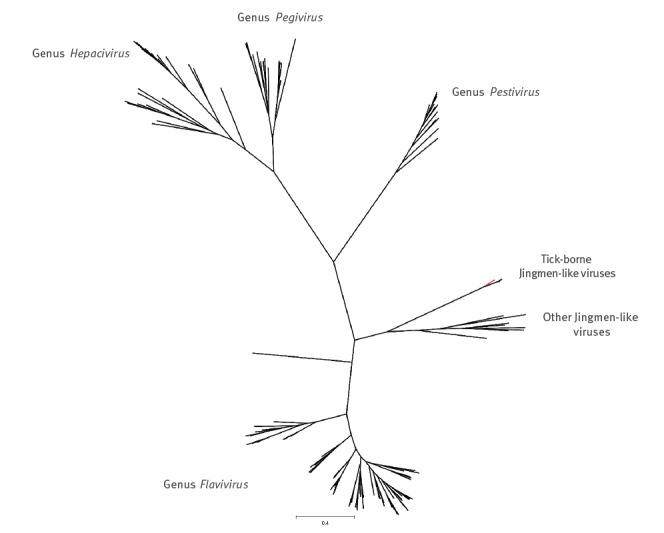
The phylogenetic tree of NS5 of all species in the family *Flaviviridae*

In 2019, while performing a metatranscriptomic analysis of ticks collected in 2011 from Haapasaari island, Kotka archipelago, south-eastern Finland, we detected a full genome of JMTV-like virus together with tick-borne encephalitis virus (TBEV) genome. Thereafter, we used RT-PCR to screen 198 *I. ricinus* ticks collected from the Kotka archipelago in 2017 and 2018 for the presence of JMTV-like RNA. We found another positive tick from a neighbouring Kuutsalo island in the Kotka archipelago, and obtained the full genome using next-generation sequencing. The viruses (GenBank accession numbers MN107153 to MN107160) cluster together with ALSV (MH158415 to MH158418) from Heilongjian Province, China, and form a cluster distinct from the other members JMTV group, including the strains found in Kosovo (MH133313 to MH133324) [2,7] (Figure 1, Figure 2, Figure 3, Figure 4, Figure 5). Nucleotide and amino acid identities between the Finnish strains and the other tick-borne JMTV-like viruses are shown in Table 1. The virus isolation trials in Vero, SK-N-SH and CRL-2088 cells were unsuccessful.

JMTV: Jingmen tick virus.

The NS3 segment amino acid sequences of all available tick-borne Jingmen tick virus-like viruses were aligned using the ClustalW algorithm. The phylogenetic tree was constructed using the Bayesian Markov chain Monte Carlo (MCMC) method, implemented in MrBayes version 3.2 [19]. The GenBank accession number of the strains sequenced in this study are MN107155 and MN107159.

JMTV: Jingmen tick virus.

The NS5 segment amino acid sequences of all available tick-borne Jingmen tick virus-like viruses were aligned using the ClustalW algorithm. The phylogenetic tree was constructed using the Bayesian Markov chain Monte Carlo (MCMC) method, implemented in MrBayes version 3.2 [19]. The GenBank accession number of the strains sequenced in this study are MN107156 and MN107160.

JMTV: Jingmen tick virus.

The putative capsid/membrane segment amino acid sequences of all available tick-borne Jingmen tick virus-like viruses were aligned using the ClustalW algorithm. The phylogenetic tree was constructed using the Bayesian Markov chain Monte Carlo (MCMC) method, implemented in MrBayes version 3.2 [19]. The GenBank accession number of the strains sequenced in this study are MN107153 and MN107157.

JMTV: Jingmen tick virus.

The putative glycoprotein segment amino acid sequences of all available tick-borne Jingmen tick virus-like viruses were aligned using the ClustalW algorithm. The phylogenetic tree was constructed using the Bayesian Markov chain Monte Carlo (MCMC) method, implemented in MrBayes version 3.2 [19]. The GenBank accession number of the strains sequenced in this study are MN107154 and MN107158.

NS5 sequences of the representatives of all flavivirus species were retrieved from NCBI Reference Sequence Database (RefSeq) and aligned with MAFFT programme version 7 [17] using E-INS-i algorithm, followed by removal of ambiguously aligned amino acid sites using TrimAl programme [18]. The phylogenetic tree was constructed using the Bayesian Markov chain Monte Carlo (MCMC) method, implemented in MrBayes version 3.2 [19].

**Table 1:** Nt and amino acid identities between Finnish strains of Alongshan virus and other tick-borne JMTV-like viruses, Finland, 2019

Segment	Nt identity (%)			Amino acid identity (%)		
	ALSV (Finland)	ALSV (China)	Other JMTV-like viruses	ALSV (Finland)	ALSV (China)	Other JMTV-like viruses
Putative capsid/membrane	5.3	9.5–9.7	33.1–37.7	1.4	2.4–2.8	29.0–36.3
Putative glycoprotein	1.5	8.0–8.1	35.9–43.1	0.5	4.4^a^	38.3–51.2
NS3	4.8	8.8–9.0	28.1–31.1	0.5	1.4–1.6	16.1–21.7
NS5	4.0	10.5–10.6	28.3–32.1	0.9	2.1–2.5	19.8–21.6

ALSV: Alongshan virus; JMTV: Jingmen tick virus; Nt: nucleotide.

^a^ Both ALSV strains from Finland have the same number of amino acid differences compared to the Chinese strain.

## Human and tick samples

The emerging reports on the association of JMTV-like viruses with human disease in China [1,2] led us to investigate sera of TBE-suspected cases for JMTV-like virus RNA or antibodies against recombinant proteins of ASLV in 2019. The sera panel included 974 serum samples from 879 individuals. These samples were originally sent for TBEV antibody testing to Helsinki University Hospital laboratory (Helsinki, Finland) from May to November 2018. All samples were tested for JMTV RNA by RT-PCR, with 304 from 283 individuals (median age: 48 years, range: 1–88 years) for antibodies to JMTV VP1a, VP1b, membrane and capsid proteins. For the RNA detection, we could verify that the RT-PCR detects local Finnish strains of ALSV, but we had no human ALSV positive control samples available for the antibody tests. We also studied three serial samples from two patients positive in an earlier sample for JMTV RNA from Kosovo at dilutions 1:20 and 1:80 for reference. These two patients shown to be infected with Kosovo strains of JMTV (capsid/membrane 63.5–64.0% amino acid identity, glycoprotein 49.9–50.1% amino acid identity) did not exhibit clear reactivity to the ALSV recombinant protein [7]. The 90 and 108 ticks collected in 2017 and 2018, respectively, from Kuutsalo island, Kotka archipelago in south-eastern Finland were tested for JMTV RNA (Table 2).

**Table 2:** Summary of JMTV-like virus RT-PCR, NGS and antibody tests of *I. ricinus* ticks and sera from suspected human TBE cases., Finland, 2019

Sample	Year	Sample origin	Number Studied (N)	Number of positives (n)	Method	Sequencing result of positive sample
*I. ricinus *panel	2011	Haapasaari island, Kotka archipelago	3	1	NGS	Whole genome ALSV
*I. ricinus *panel	2017	Kuutsalo island, Kotka archipelago	90	1	Conventional RT-PCR	Whole genome ALSV
*I. ricinus *panel	2018	Kuutsalo island, Kotka archipelago	108	0	Conventional and real-time RT-PCR	NA
Human sera from suspected TBE case	2018	Throughout Finland	974	0	Real-time RT-PCR	NA
Human sera from suspected TBE case	2018	Throughout Finland	304	0	Recombinant JMTV protein IFA	NA

ALSV: Alongshan virus; IFA: immunofluorescence assay; JMTV: Jingmen tick virus; NA: not applicable; NGS: next generation sequencing; TBE: tick-borne encephalitis.

## RNA and antibody detection

Ticks were homogenised and RNA was extracted as described previously [10]. Total nucleic acids from human serum samples were extracted using MagNa Pure LC 2.0 instrument and Total Nucleic Acid Isolation Kit (Roche, Basel, Switzerland). Viral RNA was detected with real-time or conventional reverse transcription (RT)-PCR targeting the NS5 gene. Primers and the probe were designed based on sequences available to us in August 2018, and we used JM F1312 as the forward primer (5’-TTCGGRGCMTGGCAMCTSACCT-3’), JM1548 as the reverse primer (5’-CCKGTTDTCCATYTGGTADCCCAT-3’), and JM2 as the probe (FAM-CTCCTAAAGATGTTAAACACTGC-BHQ). Conventional RT-PCR without the probe was initially used for tick samples with SuperScript III One-Step RT-PCR System with Platinum Taq DNA Polymerase (Invitrogen, Carlsbad, California, United States (US)). Patient and tick samples were screened with real-time RT-PCR using the TaqMan Fast Virus 1-Step Master Mix (Thermo Scientific, Waltham, Massachusetts, US). An in vitro transcribed RNA served as the positive control.

Synthetic gene constructs encoding JTMV glycoproteins VP1a, VP1b, membrane and capsid proteins were cloned into pCAGGS/MCS [11]. The recombinant and empty plasmids were transfected into Vero E6 cells using Fugene HD according to the manufacturer’s instructions. The transfected cells were fixed onto microscopic slides with acetone, serum samples were diluted 1:20 in phosphate-buffered saline and immunofluorescence assay was performed as described previously [12].

## Next generation sequencing and phylogenetic analysis

Tick homogenates were treated with a mixture of micrococcal nuclease (New England BioLabs Ipswich, Massachusetts, US) and benzonase (Millipore, Burlington, Massachusetts, US) for 1 hour at 37 °C, followed by RNA extraction using TriPure Isolation reagent (Roche, Basel, Switzerland). RRNA was removed using a NEBNext rRNA Depletion Kit (New England BioLabs) according to the manufacturer’s protocol. The sequencing library was prepared using a NEBNext Ultra II RNA Library Prep Kit (New England BioLabs). The library fragment sizes were measured using agarose gel electrophoresis and the concentrations using Qubit dsDNA BR Assay Kit (Life Technologies, Carlsbad, California, US) and NEBNext Library Quant Kit for Illumina (New England BioLabs). Sequencing was conducted using MiSeq Reagent Kit V2 with 150 bp reads.

Raw sequence reads were trimmed and low-quality, quality score < 15, and short, < 36 nt, sequences were removed using Trimmomatic [13]. Thereafter, de novo assembly was conducted using MegaHit [14]. Open reading frames were sought using MetaGeneAnnotator [15], followed by taxonomic annotation using SANSparallel [16].

Complete genome sequences of all available tick-borne JMTV-like viruses were downloaded from GenBank (accessed June 2019). The amino acid sequences were aligned using the ClustalW algorithm followed by manual refinement. In addition, NS5 sequences of the representatives of all flavivirus species were retrieved from NCBI Reference Sequence Database (RefSeq) and aligned with MAFFT programme version 7 [17] using E-INS-i algorithm, followed by removal of ambiguously aligned amino acid sites using TrimAl programme [18].

The phylogenetic trees were constructed using the Bayesian Markov chain Monte Carlo (MCMC) method, implemented in MrBayes version 3.2 [19] with two independent runs and four chains per run. The analysis was run for 5 million states and sampled every 5,000 steps.

## Conclusion

Our findings show that ALSV, a newly described tick-borne human pathogen, is also present in south-eastern Finland. Notably, ALSV was detected in *I. ricinus* ticks, a tick species that is common across the European continent. Despite apparent ALSV circulation in the south-eastern archipelago of Finland, no ALSV RNA or antibodies to selected recombinant ALSV proteins were found in ca 900 Finnish patients suspected for TBEV infection in recent years. While our results suggest low human infection pressure, further research using other methods, including properly evaluated ALSV antibody tests, and focusing on other geographic areas and patient cohorts beyond meningitis or encephalitis cases is needed.

## Note

*This designation is without prejudice to positions on status, and is in line with United Nations Security Council Resolution 1244/99 and the International Court of Justice Opinion on the Kosovo Declaration of Independence.
